# Quality‐assured research environments for translational cancer research

**DOI:** 10.1002/1878-0261.12441

**Published:** 2019-02-22

**Authors:** Anton Berns

**Affiliations:** ^1^ Oncode Institute The Netherlands Cancer Institute Amsterdam The Netherlands

**Keywords:** comprehensive cancer centre, EACS, prevention, research quality, translational research

## Abstract

In order to secure high‐quality cancer care for increasing numbers of cancer patients in the upcoming decades, the complete continuum of cancer research and cancer care needs a thorough overhaul, with more emphasis on prevention and early detection, and a greater focus on the development of innovative treatments that are also scrutinised for effectiveness and quality‐of‐life aspects. Therefore, under‐resourced research areas, such as primary prevention, early diagnosis/secondary prevention (Song *et al*., 2018; Wild *et al*., 2015) and outcomes research (Cavers *et al*., 2017), should be given more emphasis, whereas basic, preclinical and clinical cancer research requires more innovation and effective collaboration to develop more effective treatments at an affordable cost. Innovative collaborative research in this translational trajectory requires the participation of well‐resourced and well‐organised institutions that are committed to high scientific and ethical standards. Offering focused funding to distinct segments of this research continuum concomitant with incentives to aspire to high‐quality standards is the most effective route to achieve these goals. Therefore, a rigorous quality assessment system for institutions operating in this research continuum is a high priority.

**Abbreviations**
CCC: comprehensive cancer centre
DKH: Deutsche Krebshilfe (German Cancer Aid)
DoE: Designation of Excellence
EACS: European Academy of Cancer Sciences
HPV: human papillomavirus
ISQua: International Society of Quality in Health Care
OECI: Organisation of European Cancer Institutes

## 1 Introduction

Insights into the underlying molecular mechanisms of cancer development have provided important new inroads in treating cancer. Still, we seem to continuously underestimate the capacity of cancer cells to evade these ever more sophisticated treatments through adaptation and selection, processes so well known from Darwinian evolution. As a result, progress in effectively treating cancer has been much slower than many of us have anticipated a decade ago. To better define and understand this greater complexity and translate the resulting knowledge into effective treatments constitute a major effort. Still, we might be unable – even with the implementation of future breakthroughs – to significantly prolong the life of a sizable subset of cancer patients. This points to the necessity to also heavily embark on primary and secondary prevention (Song *et al*., 2018) as well as promote outcomes research (Cavers *et al*., 2017). The fact that cancer treatments almost invariably have substantial side effects means there is an urgent need to scrutinise the effectiveness of treatments and the quality‐of‐life consequences for patients, as well as the burden for the healthcare system, including the associated cost. This raises the question how we best organise this continuum of basic, preclinical and clinical research in order to catalyse these developments. (Ringborg, 2019). Institutions in which different disciplines closely collaborate and investigators are able to swiftly test new concepts and validate those in clinical trials seem best positioned for this. Comprehensive cancer centres already fulfil such a role by integrating basic cancer research with clinical research (including clinical trials). For research in the area of primary prevention, institutes with strong epidemiological expertise and access to large patient databases and biospecimen repositories seem best positioned. These activities might be conducted within comprehensive cancer centres or operate independently with a different set‐up and within other organisational structures. The access to and expertise to interrogate large public records make these latter entities also better suited to conduct outcomes research in which the actual benefit of treatments for patients and the associated costs can be objectively evaluated in the population.

To execute these tasks properly, institutions in any of these categories need to have critical mass, necessary expertise and adequate resources. However, even dedicated single institutions might have insufficient reach to conduct such studies without collaborating closely with other institutions with which they share technological platforms, quality standards, patient cohorts, biobanks, and patient or population databases. Furthermore, they have to collectively cover the complete trajectory of research and have the capacity to disseminate the acquired expertise to substantially improve overall cancer care. Some areas, such as prevention, early detection and outcomes research, have so far received limited emphasis and concomitantly modest funding, whereas, at the same time, certain inefficiencies and redundancies in the area of the (pre)clinical trajectory remain. The many concurrent ‘me too’ trials in immunotherapy can serve as an example. This limits innovation and, although one might expect it would lead to competition and bring down the high pricing of the resulting ‘me too’ biologicals with fairly identical mechanisms of action and effectiveness, this appears to not be the case.

## 2 How can we improve the system?

First of all, all aspects of the research continuum, namely basic cancer research, prevention, early detection, secondary prevention, translational research, clinical evaluation and outcomes research, should have access to funding mechanisms tailored to the specific needs for advancing the respective field. The knowledge and insights obtained will facilitate building a more effective cancer care system with broad access and at an affordable cost. Part of the cost‐effectiveness has to come from prevention and critical assessment of the ‘added value’ of new treatments. The European Academy of Cancer Sciences has recently published a ‘position paper’, which emphasises the areas that need attention (Adami *et al*., 2018). To support this continuum of research in a balanced fashion, some top‐down encouragements should be considered.

1. Encourage institutes and universities to invest in less‐explored aspects of the cancer research continuum and promote cross‐disciplinary interactions. This includes early detection, identification of high‐risk individuals, and primary and secondary prevention, as well as outcomes research (Calvert *et al*., 2018). Therefore, incentives have to be put in place to seduce institutions to build capacity in these areas. This will require long‐term funding streams.
2. Provide incentives to comprehensive cancer centres, universities and institutes to create sufficient critical mass and to impose high‐quality standards, and promote critical assessment of their quality by external independent audits using well‐designed EU‐wide accepted protocols. Preferentially, this should become standard for all institutions engaged in medical research. The assessments should be tailored to their specific expertise and role in the healthcare continuum.
3. Provide incentives for institutional collaboration permitting participants to effectively engage in joint large initiatives, thereby taking advantage of unique expertise present at the individual institutions. This will facilitate swift execution of innovative investigator‐initiated data‐rich studies and clinical trials of which the data sets and records will also retain value for future sophisticated AI‐based analyses. Furthermore, specific incentives to foster collaborations between institutes in rich countries in western Europe and less‐resourced countries in Europe could help these latter institutes to more quickly achieve a good quality level of cancer care.
4. Assure that proven strategies for effective prevention and treatment are swiftly implemented in the healthcare system. For preventive measures, this might be a political (smoking) or social (HPV vaccination) issue, rather than a scientific issue. It is worth reflecting how many lives would have been saved if we, as a society, had been more forthcoming in the past to discourage smoking, known to be extremely harmful for over 50 years, and had had the courage to implement measures that have now been implemented or will eventually be implemented, decades ago? In this regard, we need also to be more proactive for the runner‐up killers, such as obesity.

## 3 The role of quality‐assured research environments

Whereas society as a whole can decide to encourage a specific field of research to solve a societal problem, the success of such initiatives will depend not only on whether the field has the knowledge level and experts to conduct such research, but also whether these experts are embedded in organisational structures that enables them to deliver. Quality‐assured research environments come into play here. Fortunately, we do not have to start from scratch.

Accreditation systems are well established in the medical profession, and this has substantially contributed to the overall quality of medical care, including cancer care. However, the astounding complexity of cancer imposes specific demands on how cancer research and cancer care should be organised. During the last decade, several programmes have been initiated that have tried to define the conditions and environments that optimally foster innovative basic, translational and clinical research. Comprehensive cancer centres that bring together high‐quality basic research with translational and clinical research appear a good formula. There are now a number of national initiatives to stimulate their establishment and further development, such as those taken by the German Cancer Aid (Deutsche Krebshilfe, DKH) that has established a quality assessment programme tuned to promote an optimal interdisciplinary clinical research environment in Germany. To achieve this, DKH contributes to the development of a limited number of interdisciplinary oncology centres of excellence by a programme, which aims to establish nationwide standards for clinical research and strengthen translational cancer research. Similarly, the largest Cancer Charity in the world, Cancer Research UK, has an ongoing activity to support and evaluate cancer research centres in Britain by external site visit committees with experts in basic, translational and clinical research. Besides these national initiatives in which a diversity of organisations (government, charities and professional societies) plays a role, there are also EU‐wide organisations that have established EU‐wide standards for assessment of cancer research environments.

The Organisation of European Cancer Institutes (OECI) has launched an accreditation programme that permits cancer centres to assess their qualification as a comprehensive cancer centre (Saghatchian *et al*., 2008, 2014 Oberst 2019). The OECI programme has been a great success and has resulted in the assessment of around 50 comprehensive cancer centres in Europe. Because many centres should – with some effort – be able to meet the quality standards defined by the OECI, this programme has already substantially augmented the quality of basic, translational and clinical cancer research in institutions throughout Europe (Rajan *et al*., 2015) and now exploits an advanced e‐tool to facilitate communication between the centres and the audit team (Wind *et al*., 2018). Furthermore, the OECI accreditation programme has been recently ISQua‐certified, thereby providing international recognition for this programme.

The European Academy of Cancer Sciences (EACS), an academy initiated by a number of Nobel laureates with the aim of creating an organisation of prominent researchers and clinicians that can provide authoritative recommendations in the field of cancer research and cancer care to institutions and policymakers, is supervising a ‘Designation of Excellence (DoE) programme that has been developed within the EU‐funded EurocanPlatform project. This encompasses an evaluation procedure that assesses whether a comprehensive cancer centre exhibits exceptional quality in covering the continuum of basic, translational and clinical research (Rajan *et al*., 2016). If an institute believes it meets the requirements for this distinction, it can request that the EACS conduct an assessment as described in a detailed protocol. Upon evaluation, which includes a site visit by renowned international experts, the cancer centre can be granted the Designation of Excellence (DoE) distinction. Thus, it is not a substitute but rather an add‐on to the assessments as offered by the OECI or Deutsche Krebshilfe (DKH). Therefore, the EACS DoE demands prior accreditation by one of these organisations. The OECI, DKH and EACS will likely soon sign a memorandum of understanding in which they define the way in which they will work together, to further improve and align their own specific assessment procedures. The Designation of Excellence protocol of the EACS has been evaluated in a pilot setting in 2015 and has assessed two comprehensive cancer centres in 2017. Several other centres in Europe have indicated their interest in pursuing the DoE assessment in 2019.

## 4 What next?

Comprehensive cancer centres in Europe largely focus on basic, translational and clinical research. Prevention and outcomes research, as well as active dissemination of skills and expertise to peripheral hospitals, are often not a prominent activity in their portfolio. Prevention, early detection and outcomes research are mostly conducted outside Comprehensive cancer centres by university departments or governmental health organisations. The latter are often responsible for prevention and early detection screening, depending on the way health care is organised in a particular country. Prevention and early detection research are important drivers of reducing the burden of cancer. Similarly, critical evaluation of how new treatments work out in daily practice with attention to quality‐of‐life as well as the socioeconomic aspects is critical ingredients for equal access at an affordable price. Given the steeply increasing costs of cancer medicines, healthcare insurance providers put increasing emphasis on efficacy and real benefits for patients. Since research in primary and secondary prevention, as well as early detection and outcome, has been under‐resourced over long periods of time, it is necessary to build capacity in these areas with the aim of reducing the number of individuals that develop cancer or present with advanced disease. By putting in place new funding streams, for example in the context of a European Mission in Cancer, it should be possible to boost activities in these fields.

It is also important that such studies are conducted throughout the EU. In fact, this applies for the complete continuum of cancer research and cancer care. This requires that the research institutions (which may be comprehensive cancer centres or other organisations with sufficient critical mass and expertise focusing on prevention or outcomes research) collaborate with similar entities in other European countries. Establishing a series of dedicated collaborating institutional clusters encompassing a few participants (e.g. ~5) to keep it manageable might be a suitable approach, as advocated over a decade ago (Ringborg *et al*., 2008). Examples of such initiatives are slowly becoming apparent and deserve further encouragement. Cancer Core Europe, a legal entity of a small number of comprehensive cancer centres in Europe, is such an early‐day example (Calvo *et al*., 2018 Eggermont *et al*., 2019). A similar collaborative initiative has been taken in the area of prevention: Cancer Prevention Europe (Forman *et al*., 2018). Such clusters could jointly apply for funding to execute well‐defined studies in the cancer research continuum. The EACS could play a role in assessing such collaborative groupings through tailored assessments that include site visits by international experts, similar to what it provides for individual comprehensive cancer centres. The quality parameters it has defined for the assessment of comprehensive cancer centres can be expanded with quality parameters that specifically focus on the added value and economical gain through joining forces. Similarly, many of the parameters that apply to comprehensive cancer centres, will, with some adjustments, also apply to organisations that focus on prevention, early detection and outcomes research. Therefore, the EACS, with its members from all over Europe, could play an important supportive role in securing the objective assessment of the quality and effectiveness of the clusters focusing on these areas. This is fully in line with a position paper recently published by the board of the EACS (Adami *et al*., 2018).

In order to stimulate the quality and critical mass of the cancer research continuum in Europe, a mission in cancer could serve as an important driver. Demanding verified institutional quality parameters as a prerequisite to apply for funding of collaborative projects would serve as an important stimulus for the field to raise the bar and engage in effective collaborations. To give a flavour of the parameters that might serve to encourage quality and collaboration throughout the cancer research continuum, a summary of the items specifically addressed in the current EACS protocol for the Designation of Excellence is shown in Box 1. The institute that requests evaluation needs to provide written responses to each of the items depicted in Box 1. The items listed are complemented by a set of more specific questions and the rationale for the criterion (Rajan *et al*., 2016). After an initial check for completeness by members of the standing committee of the EACS, an international committee of renowned experts evaluates the report and further explores the strengths and weaknesses of the centre during an audit on site. It subsequently provides a detailed report that is sent to the institution for factual comments. The site visit report and the institute's comments are subsequently reviewed by a standing committee of the EACS, which then makes a recommendation to the board of the EACS whether or not to assign the Designation of Excellence distinction to the institution, or it might specify specific improvements needed to qualify for the DoE distinction. A mission in cancer (Celis and Pavalkis, 2017) – if well designed and with the right incentives – could provide a major stimulus to the cancer research field, resulting in more effective collaboration and capacity building in the continuum of cancer research and cancer care. This will be an essential component for reducing the incidence of advanced disease and more effectively treating cancer patients with progressed disease. If we fail to act, we might soon have neither the personnel nor the financial resources to offer cancer patients the care they deserve.

Box 1 1

1. Articulation of a vision of the cancer centre's philosophy, scientific directions and goals for the next 5 and 10 years; and which projects and translational science studies are expected to have real impact on the clinical oncology field.
2. Demonstration (supported by organisational data, publications and description of successfully executed original clinical trials) of a broad translational research programme of high quality in which the connection between basic research and clinical application (clinical trials) has been successfully implemented. This must address major unanswered questions in the field and unmet clinical needs.
3. Experience with and commitment to a team science approach with basic and more applied scientists working together to achieve translational goals.
4. Tangible evidence of a commitment to collaboration both within the cancer centre's own country and internationally, as a single Centre usually will be less effective in developing and testing new approaches that lead to changes in clinical practice.
5. Establishment of shared resource facilities (Cores) to support the research programmes.
6. National and international peer review systems (including evaluation by funding and government bodies) assess the Centre on a regular basis to help maintain and improve the overall quality of the programmes, leadership, shared facilities (e.g. biospecimen banks) and research/clinical studies.
7. Commitment to a programme of training of new translational scientists and retraining of established basic, clinical or population scientists who wish to redirect their careers into translational cancer research.
8. Establishment of an up‐to‐date fully and clinically annotated biospecimen bank (or banks) with an information technology system or network for tracking specimens and linkage to clinical outcome and follow‐up data. To optimise the impact of the bank, specimens should be shared with other researchers or collaborators.
9. Ability and commitment to perform hypothesis‐driven and hypothesis‐generating clinical and population studies.
10. Demonstration of a sufficiently large patient population to support bench to bedside studies in all the programmatic areas cited. Smaller cancer units should collaborate in their clinical trials in an effort to reach large enough numbers of patients to render the outcomes of these studies valid and effective.
11. Commitment to funding high‐risk/high‐reward projects to seize new and exciting research opportunities.
12. A detailed demonstration of the ongoing ability and a clearly articulated intention to leverage core funding and/or resources as a result of an ‘excellent’ designation.
13. Involvement of patient advocates in advisory committees.
14. Criteria outside the realm of criteria 1–13 that you consider important to communicate to the site visit committee or the EACS.
15. Any other issues you want to point out that you consider relevant for the designation process in general or more specific for your centre.

## Conflict of interest

The author has no conflicts of interest to report.
